# Lateral T-Capsulotomy with Hip Arthroscopy for Access and Excision of Posterior Intra-Articular Mass

**DOI:** 10.1016/j.eats.2022.08.004

**Published:** 2022-10-20

**Authors:** Rami G. Alrabaa, Abhishek Kannan, Ajay S. Padaki, Alan L. Zhang

**Affiliations:** Department of Orthopaedic Surgery, University of California–San Francisco, San Francisco, California, U.S.A.

## Abstract

There is a lack of literature regarding arthroscopic access to the posterior peripheral compartment of the hip. Compared with open surgery, arthroscopy offers less-invasive treatment for intra-articular mass excision. Arthroscopic hip mass excision has focused on selective resection of lesions in the central compartment and anterior peripheral compartment due to difficult and previously undescribed posterior access. We introduce a technique for arthroscopic excision of a posterior intra-articular hip mass consistent with pigmented villonodular synovitis, also known as tenosynovial giant cell tumor, using a modified T-capsulotomy based on the lateral aspect of the hip capsule. This modified capsulotomy allows for more posterior and lateral access to the central and peripheral compartments while minimizing violation of the iliofemoral ligament.

Clinical presentation of pigmented villonodular synovitis (PVNS), also known as tenosynovial giant cell tumor, is nonspecific, seen most commonly in patients in the second to fifth decades of life with pain as a primary symptom.^1^ PVNS localized to the hip joint may present as sudden onset of pain, limited range of motion, or sensation of joint fullness in an otherwise-healthy, active individual without previous injury or ailment affecting the lower extremities. PVNS has been reported as a mimicker of acute septic arthritis of the hip in a young patient.^2^ In older patients, pain symptoms have been reported as only transient, presenting as limited range of motion and pain with both activity and at rest.^3^

Following clinical assessment, radiographs and magnetic resonance imaging (MRI) may be performed to further elucidate the pathology. Erosive changes seen on radiographs and MRI can lead to the potential diagnosis of PVNS with characteristic villous proliferation and hemosiderin deposits.^4^

While minimally symptomatic patients may be treated conservatively, open and arthroscopic resection combined with radiotherapy have demonstrated successful symptomatic resolution.^5^ Recurrence following partial arthroscopic synovectomy has been reported to be less than 10%, with significant alleviation of symptoms.^6^ Although arthroscopic treatment for hip PVNS offers less-invasive treatment, previous treatment has focused on selective anterior intra-articular resection due to difficult and previously undescribed posterior access to the hip joint.^7^ Previous technique descriptions offer optimization pearls for central access,^8^ yet access to the posterior capsule and posterior peripheral compartment has not been previously described. In this Technical Note, we highlight an arthroscopic approach to the posterior hip capsule for the excision of a posterior PVNS mass (Video 1).

## Surgical Technique (With Video Illustration)

**Fig 1:**
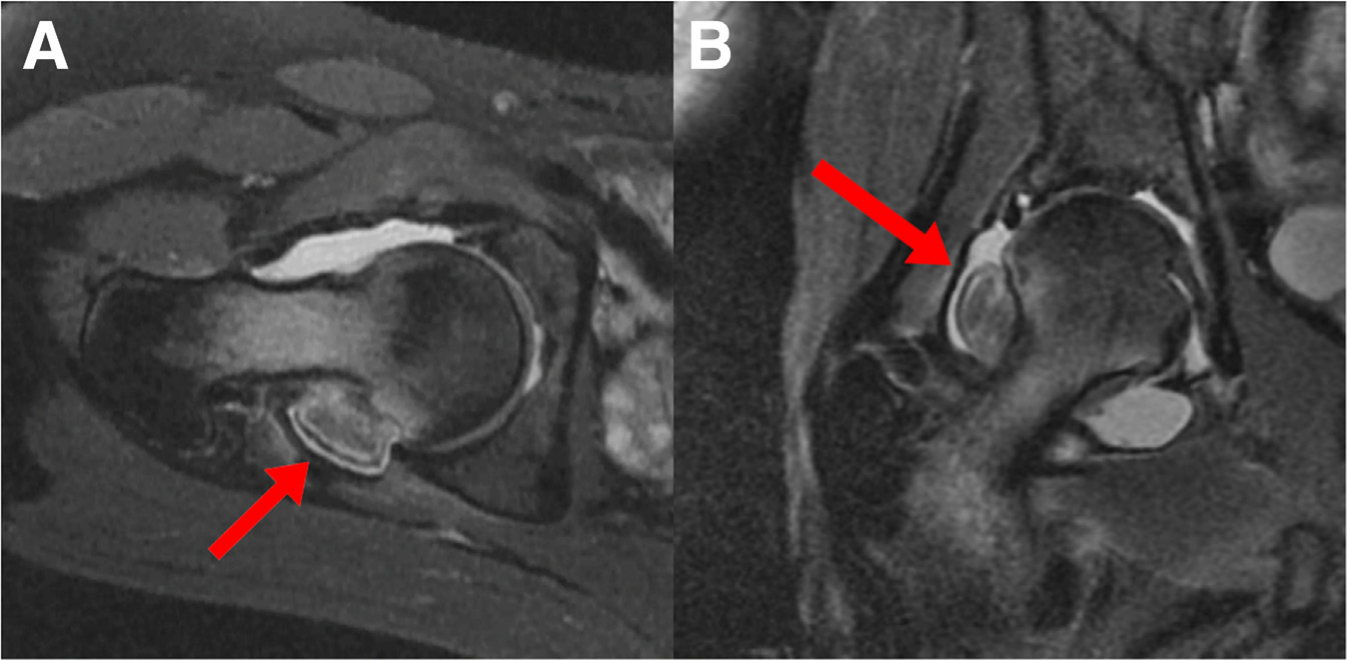
Preoperative T2-weighted magnetic resonance imaging of the right hip is shown. (A) Axial images of the right hip show the intra-articular mass (red arrow) at the posterior aspect of the femoral head-neck junction. (B) Coronal images of the right hip also show the position of the intraarticular mass (red arrow) along the posterior and lateral aspect of the femoral head–neck junction.

Preoperative MRI shows an intra-articular mass of the right hip along the posterior femoral head–neck junction (Fig 1). The patient is positioned supine on a table that allows for hip traction and for dynamic limb positioning. The operative limb is prepped and draped, an air arthrogram is performed under fluoroscopic guidance, and traction is applied. A standard anterolateral portal (ALP) is established off the anterolateral border of the greater trochanter, followed by a midanterior portal, which is created about 3 cm anterior and just distal to the ALP. A 70° arthroscope (1588 AIM; Stryker Endoscopy; Kalamazoo, MI) is used to view from the ALP for diagnostic arthroscopy. Arthroscopic management of intra-articular hip pathology is then performed as needed. Pincer lesions are resected if present and labral repair is performed if needed. Once central compartment pathology is addressed, attention is turned to access the posterior aspect of the hip joint.

**Fig 2:**
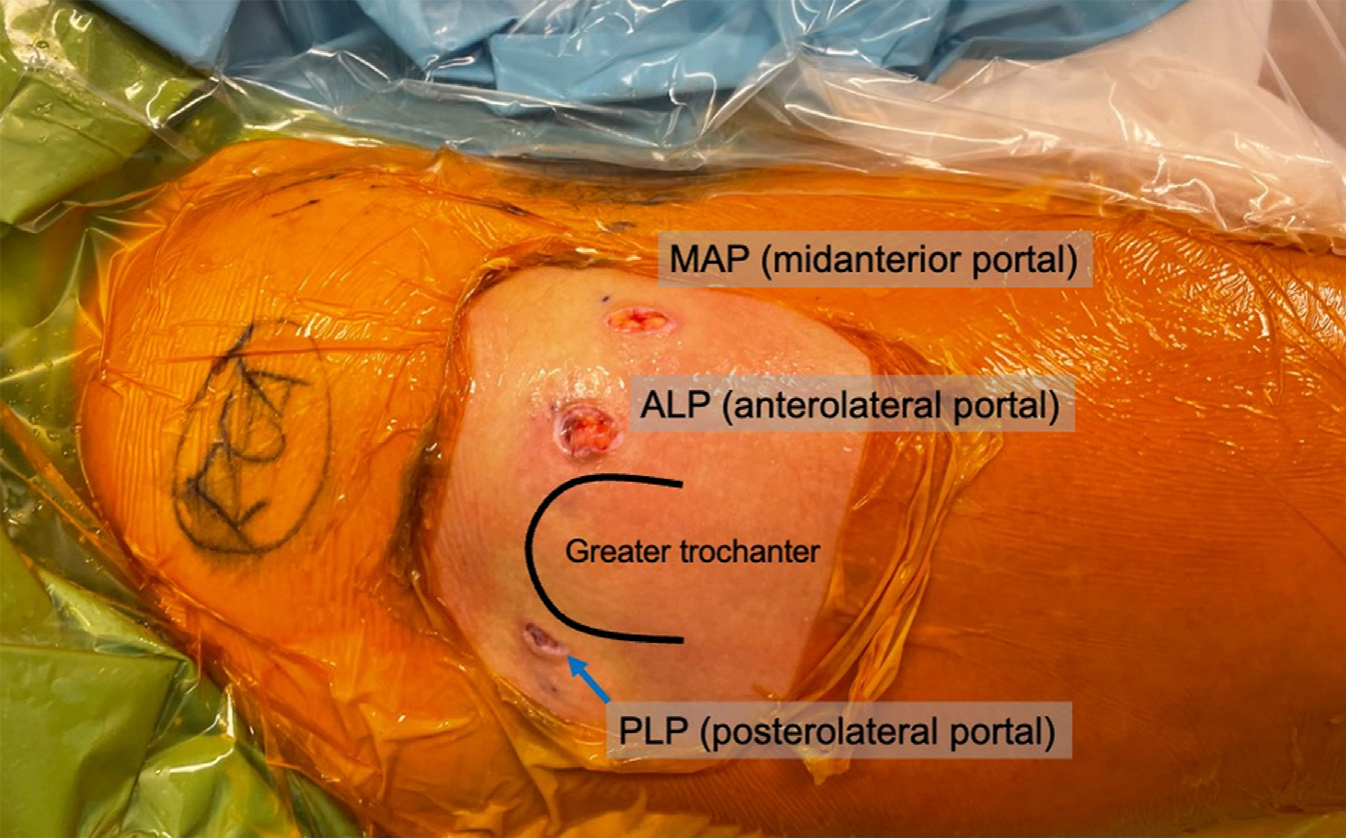
The lateral aspect of the patient’s right hip is shown as the patient lays supine for hip arthroscopy. The outline of the greater trochanter is marked and the 3 arthroscopic portals used for this technique are shown and labeled. The location of the PLP (posterolateral portal) is marked with a blue arrow just of the posterolateral aspect of the greater trochanter.

**Fig 3:**
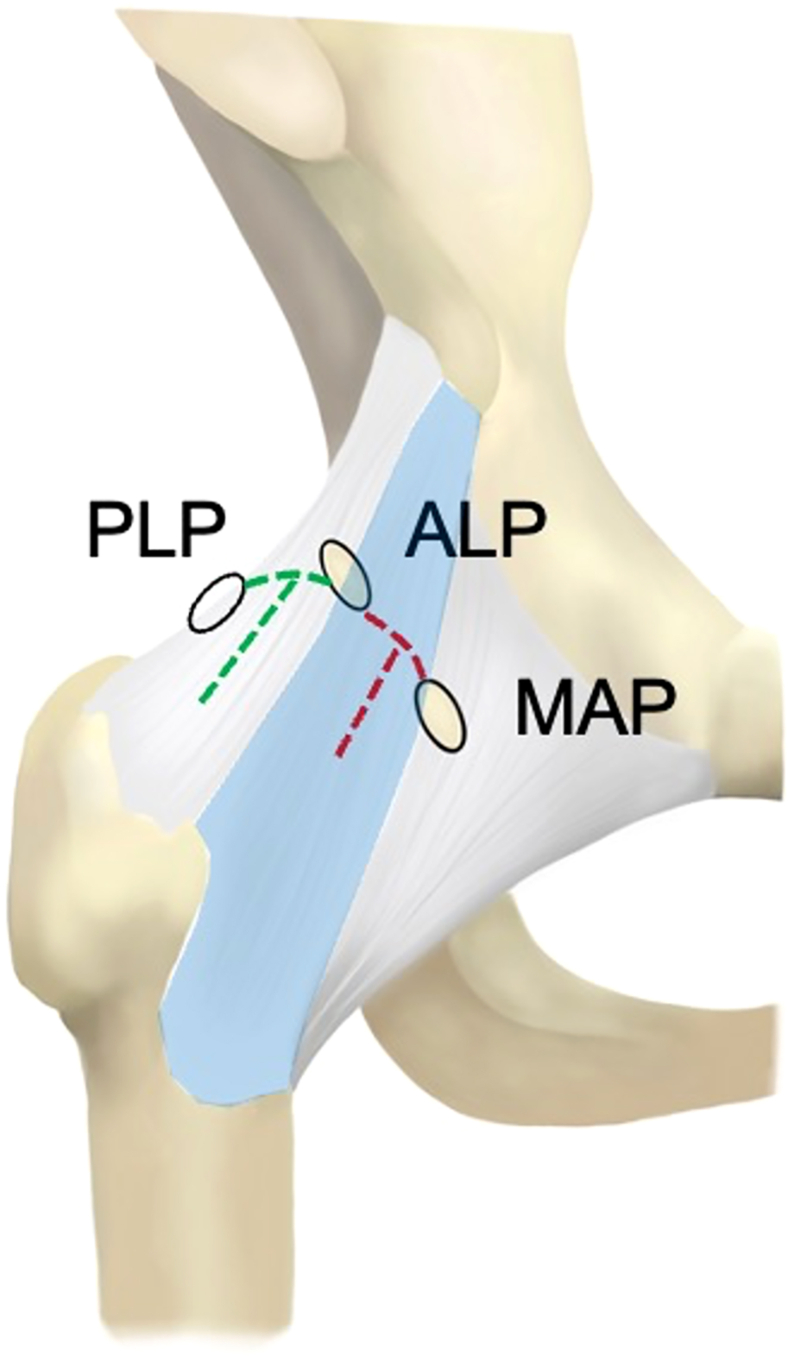
This illustration is an anterior view of a right hip showing the bony anatomy and hip capsule. The PLP, ALP, and MAP portals for hip arthroscopy are shown as ovals over their location on the hip capsule and labeled. A classic T-capsulotomy for hip arthroscopy is depicted by the red dashed line as the transvers portion of the capsulotomy is between the MAP and ALP. The modified lateral based T-capsulotomy used in this technique is depicted by the green dashed line as the transverse portion of the capsulotomy is between the ALP and PLP. This modified T-capsulotomy avoids violating the iliofemoral ligament which is highlighted in light blue. (ALP, anterolateral portal; MAP, midanterior portal; PLP, posterolateral portal.)

First, a posterolateral portal (PLP) is established while the hip is distracted. The entry point on the skin is at the posterolateral aspect of the greater trochanter (Fig 2). Intra-articular access is established with fluoroscopic and arthroscopic guidance while visualizing through the ALP with a 70° arthroscope. After the PLP portal is established, the arthroscope is switched to view through the PLP and a modified laterally based T-capsulotomy is performed. An arthroscopic blade (Samurai Blade; Stryker; Kalamazoo, MI) is used to create the capsulotomy using the ALP as the working portal. First, a transverse medial-to-lateral interportal capsulotomy is performed with the blade between the anterolateral and posterolateral portals while viewing from the PLP. After creating the transverse limb of the capsulotomy, traction can be released for the hip if central compartment access is no longer needed. Next, the longitudinal limb of the T-capsulotomy is made from proximal to distal along the axis of the femoral neck using the blade through the ALP. In a classic T-capsulotomy, the transverse portion of the capsulotomy is between the midanterior portal and the ALP. In our modified lateral T-capsulotomy, the transverse portion is between the ALP and the PLP (Fig 3). This lateral based T-capsulotomy allows for more posterior access along the femoral head–neck junction to access the mass and also avoids violating the iliofemoral ligament, which is vital for the structural integrity of the hip capsule.

**Fig 4:**
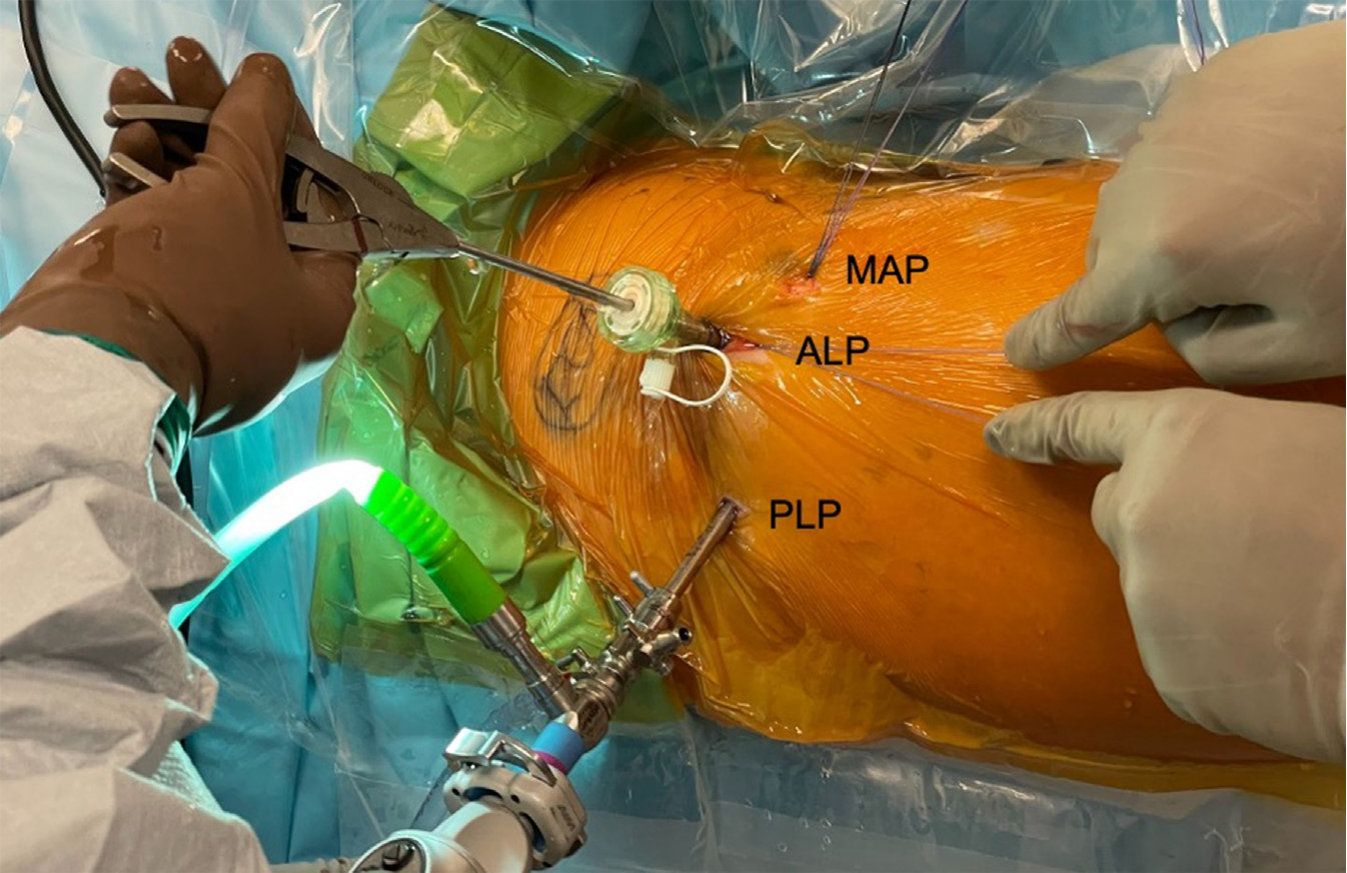
This image shows the lateral aspect of the right hip as the patient lays supine for hip arthroscopy. The 70° arthroscope is viewing from the PLP and a tissue grasper is being used from the ALP to access the intraarticular mass in the posterior femoral head-neck junction. The surgeon’s assistant is pulling traction on sutures placed through capsular leaflets after the capsulotomy in order to aid in visualization. Traction sutures can be seen exiting the ALP and MAP. (ALP, anterolateral portal; MAP, midanterior portal; PLP, posterolateral portal.)

**Fig 5:**
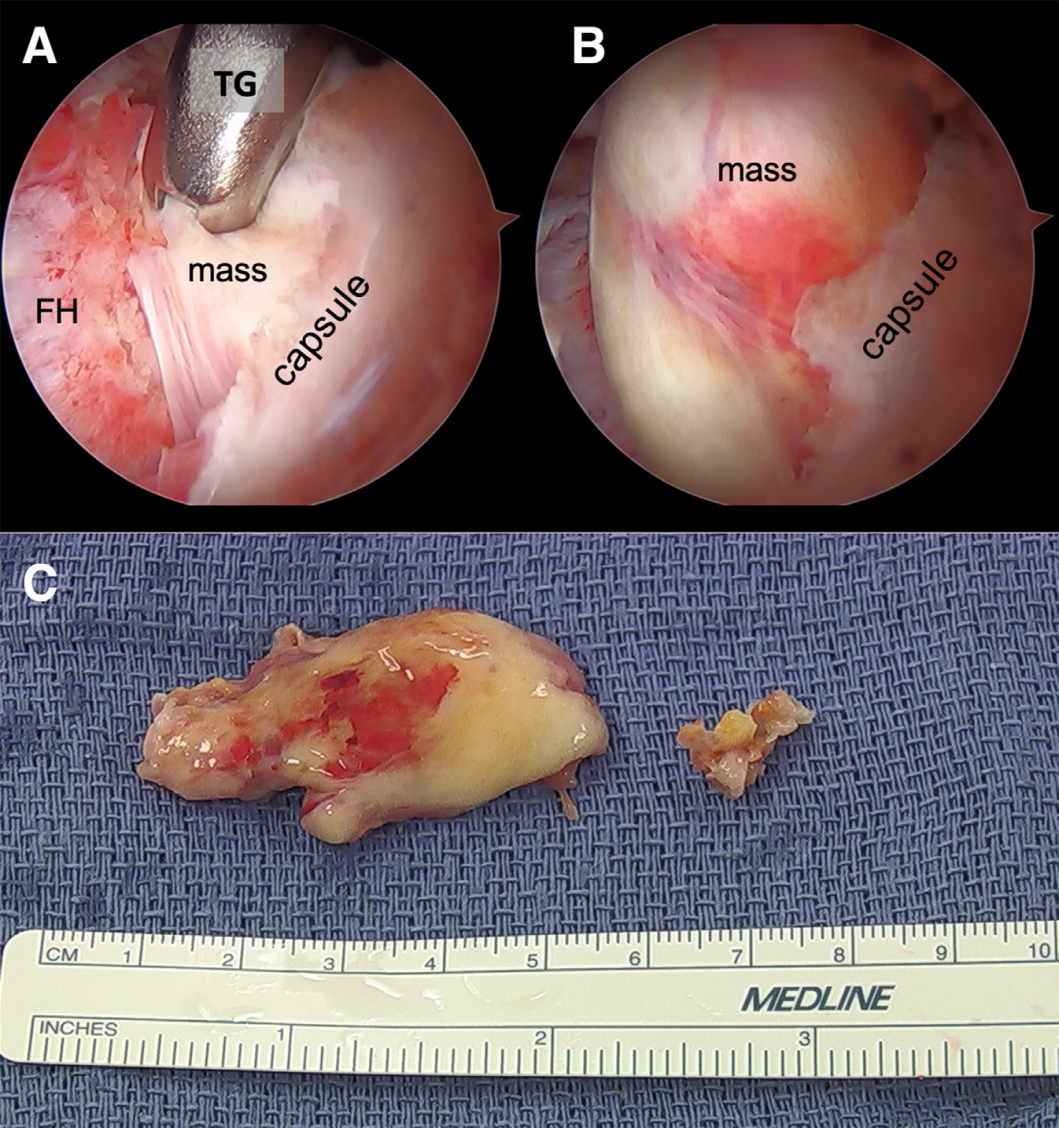
(A) Arthroscopic view of a right hip viewed with a 70° arthroscope from the PLP with an arthroscopic TG introduced from the ALP accessing a posterior intraarticular mass along the posterior femoral head–neck junction. The mass (labeled) is grasped deep to the capsule (labeled) and with gentle traction and rotation the mass is separated from the capsule. (B) As the mass is pulled with the grasper it comes more in view. (C) It is ideal to excise the mass in one whole segment. (ALP, anterolateral portal; FH, femoral head; PLP, posterolateral portal; TG, tissue grasper.)

**Fig 6:**
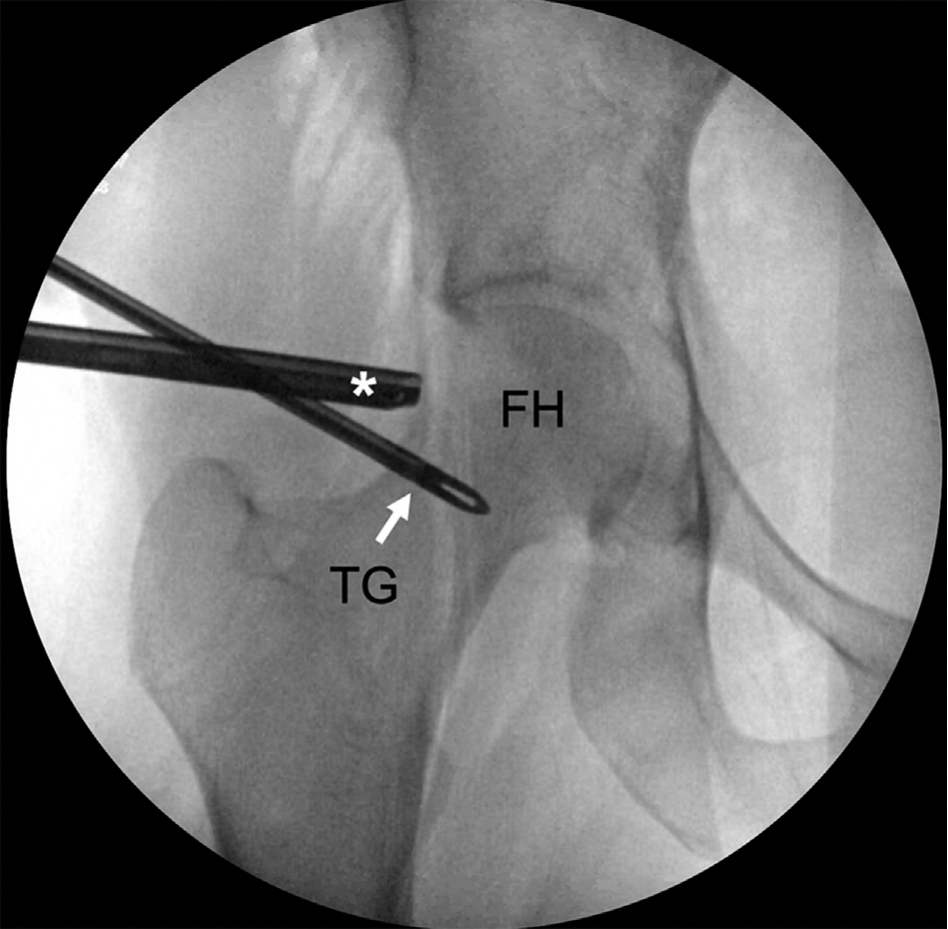
Intraoperative anteroposterior fluoroscopic image of the right hip during hip arthroscopy. This intraoperative image depicts the extent of the posterior and distal access along the femoral neck that was achieved with the TG with this technique. The 70° arthroscope (∗) is viewing from the posterolateral portal and the TG is being used from the anterolateral portal. (FH, femoral head; TG, tissue grasper.)

After the modified T-capsulotomy is made, traction sutures can be placed in the leaflets of the capsule to aid in retraction while accessing the peripheral compartment. A disposable 8-mm cannula or a metal sled can be used for introducing instruments through the ALP working portal. The 70° arthroscope is used to view from the PLP and an arthroscopic tissue grasper is introduced from the ALP to access the posterior aspect of the femoral head–neck junction (Fig 4). The tissue grasper is used to securely grasp the mass from underneath the capsule and with gentle traction and rotation to excise the mass (Fig 5). It is ideal to remove the entirety of the mass in one whole segment. Intraoperative fluoroscopy shows the extent of the posterior and distal access that can be achieved with this technique (Fig 6).

After the mass is excised, a synovectomy is performed in the area adjacent to the mass. Hemostasis also is ensured with the use of a radiofrequency ablation device. Finally, closure of the T-capsulotomy is performed. A 70° suture passer (SlingShot; Stryker) is used to pass #2 high tensile strength suture (ORTHOCORD; DePuy Synthes Mitek; Raynham, MA) through the capsular leaflets for capsular closure. Three interrupted simple sutures are used to close the T-capsulotomy with one suture in the longitudinal limb and 2 sutures in the transverse limb.

Advantages and disadvantages (Table 1) as well as pearls and potential pitfalls (Table 2) of this technique are summarized.

**Table 1:** Advantages and Disadvantages of Using a Lateral T-Capsulotomy for Arthroscopic Access and Excision of a Posterior Hip Mass

Advantages	Disadvantages
• Arthroscopic excision is minimally invasive compared with open surgery.	• The technique can be challenging for surgeons not familiar with hip arthroscopy.
• Increased access to the posterior peripheral compartment of the hip compared to classic anterior-based capsulotomy techniques	• T-capsulotomy requires capsular repair, which can be technically demanding and time consuming.
• The modified T-capsulotomy is more laterally based and therefore avoids violating the iliofemoral ligament.	• Inadequate capsule closure may result in instability or microinstability of the hip
	• Lateral T-capsulotomy may not offer enough access to the peripheral space inferior to femoral head.

**Table 2:** Pearls and Potential Pitfalls of Using a Lateral T-Capsulotomy for Arthroscopic Access and Excision of Posterior Hip Mass

Pearls	Potential pitfalls
• View from posterolateral portal with a 70° arthroscope.	• If longitudinal limb of T-capsulotomy is extended too distally then vascular supply to the femoral head may be compromised.
• Use anterolateral portal as main working portal.	• A blade or knife is used for the capsulotomy as opposed to a radiofrequency ablation device which can lead to bleeding.
• Establishing the posterolateral portal while the hip is still in traction allows for safe placement under direct visualization.	• Too much damage or shaving of capsular tissue can lead to deficient capsular tissue for closure.
• Intraoperative fluoroscopy can be used to aid in localization and to interpret how far posteriorly and distal the instruments are accessing.	• Insufficient capsular closure can lead to potential for postoperative hip instability.
• Clear bursal tissue overlying lateral hip capsule to better visualize capsulotomy.	
• Place traction sutures in the capsular leaflets after the capsulotomy to aid in retraction of the capsule and improve visualization.	
• Do not extend longitudinal limb of T-capsulotomy distal to zona orbicularis to protect the vascular supply.	

## Discussion

The literature on PVNS or tenosynovial giant cell tumor of the hip remains limited to case reports and cases series. Historically, open total synovectomy and conversion to hip arthroplasty was described in the treatment of hip PVNS.^9^, ^10^, ^11^ Gondolph-Zink et al.^12^ described a semiarthroscopic synovectomy of the hip, which enabled radical synovectomy without risk of femoral head necrosis. Janssens et al.^13^ described the role of diagnostic hip arthroscopy in the management of hip PVNS. While numerous authors have reported the efficacy of arthroscopic synovectomy for treatment of PVNS of the knee,^14^, ^15^, ^16^, ^17^ literature on outcomes following arthroscopic synovectomy of the hip remains limited.^7^^,^^18^, ^19^, ^20^

Byrd et al.^19^ reported on 13 patients treated for diffuse, nodular, and combined PVNS of the hip and provided outcomes at a mean of 63 months. Their cohort demonstrated significant improvements in Harris Hip Score, and patients without labral tears did have significantly better improvement postoperatively when compared to patients with labral tears.^19^

While general sentiment is that the nodular form of PVNS may respond more favorably to excision as compared with the diffuse form, studies have failed to corroborate that notion.^21^^,^^22^ Mankin et al.^21^ noted a greater failure rate of treatment for PVNS of the hip (11 of 12 patients converted to total hip arthroplasty), postulating limited capacity within joint capsule and lesion progression may contribute to periarticular destruction before clinical presentation.

While an arthroscopic approach to hip PVNS offers many advantages, it may not prove reasonable or effective for diffuse PVNS. In addition, nodular lesions localized to specific regions of the joint, such as directly inferior to the femoral head, may prove more challenging to access arthroscopically, and may be best and most safely addressed via open excision. We describe a T-capsulotomy over the lateral aspect of the hip capsule to access the posterior aspect of the hip joint for excision of nodular hip PVNS. The standard, traditionally described T-capsulotomy increases mobility of instruments, enabling a range of access from 180° to 270° around the femoral head and neck.^23^ The traditionally described T-capsulotomy is based between the mid-anterior and anterolateral portals. By centering the longitudinal limb of our T-capsulotomy on the lateral aspect of the femoral neck, between the standard anterolateral and posterolateral portals, we are able to reach the posterior aspect of the femoral neck and joint. By accessing the posterior compartment as described, additional foci of PVNS may be safely resected and possibly decrease overall recurrence rates, although further longitudinal research is required.

## Supplementary Data

ICMJE author disclosure forms

Video 1 This technique video shows the case of an arthroscopic excision of an intra-articular posterior right hip mass in a healthy female patient. The 3 portals used for include the anterolateral portal (ALP), midanterior portal (MAP), and posterolateral portal (PLP). The ALP and MAP are used for any central compartment work, including Pincer resection and labral repair. The ALP and PLP are used for excision of the posterior intraarticular mass. To access the posterior intraarticular mass, a modified lateral T-capsulotomy is made with the transverse limb of the capsulotomy made between the ALP and the PLP (as opposed between the MAP and ALP in a standard T-capsulotomy). Viewing from the PLP with a 70° arthroscope, an arthroscopic tissue grasper is used from the ALP, which can reach fairly posterior and distal along the posterior femoral neck. With traction and rotation, the grasper is used to excise the mass in one whole piece. A shaver is then used to complete a synovectomy in the area adjacent to the mass. The capsulotomy is repaired with three interrupted simple sutures.
